# Chronic Hepatitis B: Management Challenges in Resource-Poor Coun-tries

**DOI:** 10.5812/kowsar.1735143X.757

**Published:** 2011-10-01

**Authors:** Sylvester Chuks Nwokediuko

**Affiliations:** 1 Department of Medicine, University of Nigeria, Teaching Hospital, Ituku Ozalla, Enugu, Nigeria

**Keywords:** Hepatitis B, Chronic, Disease Management, Public Health

## Abstract

Sylvester Chuks Nwokediuko, Department of Medicine, University of Nigeria Teaching Hospital, Ituku OzallaChronic infection with hepatitis B virus (HBV) is a global public health problem because of its worldwide distribution and its potential to cause sequelae. HBV is most prevalent in China, South East Asia, sub-Saharan Africa, and the Amazon basin of South America where health care resources are most limited. Numerous challenges exist for effective management of chronic HBV infection, particularly in resource-limited regions. These challenges include lack of accurate prevalence data, absence of a surveillance program, and poor political will of governments in resource-poor countries to enforce effective measures to control the disease. There is a lack of understanding regarding HBV infec-tion by both the general public and health care providers. A better understanding of the pathogenesis and treatment of this condition is necessary. The acute shortage of trained medical manpower necessary for accurate diagnosis and treatment of chronic hepati-tis B (CHB) in resource-poor countries is a formidable challenge. The condition is com-plicated by the continuing efflux of medical graduates from low-income economies to richer countries. The most critical problem in the management of CHB is the high cost of laboratory tests and drugs. Drugs are also not readily available. Other challenges in the manage-ment of CHB include stigmatization of patients, co-infection with other viruses, lack of management guidelines, and absence of an effective patient referral system. To address these challenges, governments of resource-poor nations must be committed to budg-etary allocation for the implementation of health programs. It is necessary to provide awareness campaigns, health education, proper screening of blood and blood products for transfusion, active screening, intensification of existing childhood immunization, technical and financial assistance from wealthier nations, and implementation of the recommendations outlined in the Global Hepatitis Policy (2010).

## 1. Introduction and Epidemiology

The hepatitis B virus (HBV) has infected approximately 2 billion people worldwide, which represents one-third of the world population. Of these cases, 350 million (5– 7% of the world population) are chronically infected [1]. Life-threatening liver disease (cirrhosis, liver failure, and hepatocellular carcinoma [HCC]) occurs in as many as 40% of patients with hepatitis B. This infection is the 10th leading cause of death worldwide, resulting in 1.2 million deaths per year. HCC is now the 5th most prevalent cancer worldwide, causing up to 500,000 deaths each year [1]. Northern Europe, North America, and Australia have low HBV endemicity (population prevalence, < 2%); sub-Saharan Africa and the Far East have high endemicity (population prevalence, > 8%); Eastern and Southern Europe, South America, and the remaining regions in Africa and Asia have intermediate endemicity (population prevalence, 2–8%) [2]. The continent of Africa encompasses a wide geographic area with a very large population and low-income economics. At least 65 million individuals with chronic HBV (CHB) infection live in Africa [3][4]. In high endemic areas of Africa and Asia, most HBV infections occur in the first 5 years of life. Perinatal transmission predominates in East and South-East Asia; in Africa, most HBV transmissions occur before the age of 5 years through close contact within households, medical procedures, traditional scarification, and other unidentified mechanisms [5][6]. The vertical transmission rate may be lower in Africa than in Asia, partly because of a lower prevalence of hepatitis B e antigen (HBeAg) in African individuals, a major determinant of perinatal transmission [7][8]. Nigeria is a sub-Saharan African country with a population of approximately 150.7 million. The population grows by 2.3% annually, the gross domestic product (GDP) is 173.0 billion US dollars, the GDP per capita is 1,118 US dollars, GDP growth is 5.6%, life expectancy is 47.9 years, infant mortality is 85.8 per 1000 live births, and the literacy rate of women aged 15–24 years is 64.6% [9]. It is the largest black nation in the world. The number of people with HBV infection in Nigeria is unknown. However, there have been numerous prevalence studies in various populations in Nigeria. Over 70% of the population shows evidence of past infection with the virus and 7.3–24% of the population has serological evidence of current infection (average 13.7%). Thus, 19 million Nigerians are currently infected and approximately 5 million will die of causes related to HBV infection [10]. In Nigeria, HBV infection has been studied in several populations [11][12][13][14][15][16][17][18][19][20][21] (Table). Despite the availability of a safe and effective vaccine since 1982 [22][23] and its inclusion in Nigeria’s national immunization program in 1995, the vaccine only became available to the public in 2004 [24],[25]. Because of this late entry and absence of a national HBV surveillance program, the burden of hepatitis B remains substantial. Most HBV infections in Nigeria occur by horizontal transmission [26][27] as well as through blood transfusion [27][28]. A recent study showed that the median overall risk of becoming infected with HBV from a blood transfusion in sub-Saharan Africa was 4.3 per 1000 units [29]. This is a substantial number, as it translates to 28,595 new HBV infections each year. A publication by the US Center for Disease Control[30] and a study in Nigeria [31] implicated injection from untrained community medical providers, body scarification, sharing of sharp body-piercing instruments, sharing of toothbrushes, and uvulectomy as reasons for the spread of HBV [16]. A relatively high prevalence was demonstrated in a group of Nigerian female civil servants, a finding thought to be related to female circumcision. Studies from Northern Nigeria indicate that unprotected sex also plays a role in HBV transmission [21][32]. HBV has evolved into at least 8 recognized genotypes, A–H [33][34][35]. Extensive investigation of HBV genotypes in Nigeria [36][37] showed that 95% of cases belong to genotype E. Analysis of HBV  ubspecies from 2 isolated rural communities in Nigeria revealed that individuals were infected with many different HBV variants [38]. Among the dominant HBV/E strain, variant sharing was extensive, suggesting a very complex pattern of transmission. Numerous HBV strains may have been maintained within the population through socio-cultural practices such as scarification marks, sharing of sharp instruments for body piercing, and the use of non-sterile medical and surgical instruments. These practices have also been associated with HIV transmission in Nigeria [39][40]. It has been hypothesized that during the 1967–1969 WHO smallpox and measles eradication program in West and Central Africa by arm-arm injections using jet injectors, significant HBV transmission may have also occurred [41][42][43]. Such mass vaccination campaigns are as-sociated with the dissemination of HBV/E in this region of Africa [41][44]. In addition, such massive introduction and the relatively recent expansion of HBV/E in Africa indicate a significant shift in HBV/E epidemic dynamics in Africa over the past century [38]. In the past decades, substantial improvement in the understanding of HBV biology, host immune responses, and natural disease course, combined with the recent availability of highly sensitive HBV DNA assays and the advent of effective antiviral drugs with different mechanisms of action, have led to better therapeutic strategies for treating CHB infection. Given the high endemicity of HBV infection, African countries have the greatest opportunity to benefit from these advancements. Unfortunately, these countries have not taken advantage of these opportunities. The objective of this review is to highlight the challenges in the management of CHB infection in Africa, using the situation in Nigeria as an example.

**Table:** Prevalence of HBsAg in Various Nigerian Populations

	**Population Studied**	**Prevalence, %**
Fakunle et al. (1977) [13]	HCC a	49
Oli et al. (1980) [18]	DM a	9.0 b
Abiodun et al. (1985) [11]	Blood donors	10
Otu et al. (1987) [20]	Liver cirrhosis, HCC	49
Bada et al. (1996) [12]	Pregnant women	16
Olubuyide et al.(1997) [19]	HCC	59.3
Ndububa et al.(2001) [15]	HCC	61
Sirisena et al. (2002) [21]	Urban population	10.3
Mustapha et al. (2004) [19]	HCC	64.7
Nwokediuko et al. (2011) [17]	HCC	61.5
Nwokediuko et al. (2011) [16]	Civil servants	7.6

^a^ Abbreviations: DM, diabetes mellitus; HCC, hepatocellular carcinoma

^b^ v.s 2.9 in control group

## 2. Lack of Accurate Statistics Regarding the Burden of HBV Infection

CHB infection is largely asymptomatic; thus, accurate assessment of its prevalence is not possible unless all members of a population are screened. The HBV disease burden as outlined by the WHO is sufficiently startling to prompt a public health response. Mortality estimates are conservative and based on cancer incidence from registries [45]. In Africa, there are few high-quality registries; and the majority of them only record cases in cities, whereas hepatitis B prevalence is often highest in rural areas. The extent of the CHB situation in African countries was conveyed in the Global Policy on Viral Hepatitis published in 2010 by the World Hepatitis Alliance (WHA). This policy is the product of a study conducted by the WHA on behalf of the WHO across all 193 member states of the WHO. The study examined policies and programs aimed at preventing and controlling viral hepatitis and areas in which the WHO may be able to assist [46]. As revealed by the report, disease surveillance for hepatitis B is present in a  total of 82% of countries in the world. This ranges from an average of less than 60% in Africa and South-East Asian regions to 90–100% in the Americas, Europe, and the Eastern Mediterranean [46]. Sixty nine percent of the population of low-income countries live in areas that report no provisions for the surveillance of hepatitis B infection in any form. In Nigeria, there is practically no surveillance program in place for HBV infection. In 71% of countries in Africa, no prevalence estimates are available [46]. Accurate data on the prevalence of hepatitis B are extremely difficult to obtain in Nigeria and most African countries.

## 3. Public Awareness and Education

Most patients with CHB have few or no symptoms; thus, these individuals remain undiagnosed. Detection of asymptomatic individuals requires active screening programs. In low-prevalence areas of the world such as the USA, specific groups of individuals are considered to be at high risk of infection and are, therefore, recommended for screening. These include persons born in endemic areas, men who have sex with men, injecting drug users, dialysis patients, HIV-infected patients, pregnant women, families/households of infected persons, and individuals who have sexual contacts with infected persons [47]. For areas of high endemicity such as Nigeria, every individual is at risk because he/she is born or lives in a highly endemic area. Awareness among the general population is quite low in Nigeria as well as in most countries in sub- Saharan African. Of greater concern is the lack of proper education and awareness among health care providers, including doctors. This is because CHB is a disease that requires adequate  explanation, counseling, and individualized assessment for successful anti-HBV therapy. The Society for Gastroenterology and Hepatology in Nigeria identified this lack of knowledge among health care providers and took a positive step towards addressing the situation by publishing the first edition of a treatment guideline for Hepatitis B and C in Nigeria in 2009 [10]. The circulation of this document needs to be widened so that doctors and other health workers will begin to implement the guideline provisions. Stigmatization is a formidable problem in the management of CHB in Africa. There is a significant amount of ignorance in the populace regarding the causes, transmission, and clinical course of HBV infection. Many people believe that there is no treatment for HBV infection. The stigma of being recognized as a HBV-infected person may provoke a feeling of shame in the community. In Nigeria, there have been cases of undergraduates being sent away from school by university authorities because they tested positive for hepatitis B surface antigen (HBsAg). This author has had reason to intervene in such cases by writing to the authorities of the universities and educating them on HBV infection, what causes it, how it is transmitted, the implications of a positive HBsAg test, and that an HBsAg- positive student does not constitute a health risk to fellow students. Transmission requires exchange of body fluids or very close and intimate physical contact such as sexual intercourse. There have also been cases of job loss and other forms of discrimination against individuals with HBV infection. These rights abuses often occur in underdeveloped societies where the victims lack the capacity to seek redress; and even if they attempt to seek redress, the cost of justice are typically not within their reach. Another challenge for Nigerian patients is that they do not understand the phases of CHB infection and that not all phases require immediate treatment. Even after explanation, many patients find it difficult to understand   why an infection should not be treated as soon as it is detected without waiting for clear indications for treatment. Physicians typically spend a significant amount of time attempting to increase the patient’s understanding regarding this point. Occasionally, patients suspect a lack of understanding of the disease by the doctor.

## 4. Personnel

The doctor-to-population ratio in Nigeria is 3 per 10,000, compared to that of the USA which stands at 26 per 10,000. There is an acute shortage of gastroenterologists, hepatologists, and anatomical pathologists in Nigeria. The country has a population of over 150 million, but has less than 40 hepatologists to care for patients with CHB (estimated to be approximately 19 million in Nigeria). One factor responsible for this situation is what is known as the “brain drain”. An estimated 13,272 physicians trained in Sub-Saharan Africa are practicing in Australia, Canada, the UK, and the USA [48]. Approximately one-third of medical graduates from Nigerian medical schools migrate within 10 years of graduation to Canada, the UK, and the USA [49]. The small number of gastroenterologists/ hepatologists in Nigeria practice in large cities, while most patients with CHB live in rural areas. Liver biopsy is a critical procedure in the evaluation of patients with CHB. Information obtained from this examination is very useful in making decisions regarding treatment as well as determining prognosis. The number of doctors trained in this procedure, and more importantly, the number of specialists trained to interpret the findings is very low in Nigeria. Newer modalities of investigation that can be used to non-invasively diagnose liver fibrosis are not yet available in most African countries, and where they are available, most physicians can only differentiate mild fibrosis or no fibrosis from severe fibrosis [50].

## 5. Referral System

An effective referral system for patients does not exist in most African countries. In Nigeria, most people with CHB live in rural areas where facilities for performing simple tests for CHB are not available. Health facilities in rural areas are not capable of conducting the HBsAg test. Even when patients manage to undergo the test and are given a positive result, the attending health workers may not be adequately informed regarding the implications of a positive test and subsequent steps to follow. Some believe that there is no treatment for HBV infection. Most patients lack the financial resources to attend larger hospitals located in cities for proper evaluation. Even when the finances are available, they may not appreciate the need for a more aggressive search for a solution because they are largely asymptomatic. This explains why most Nigerian patients with HBV infection present with decompensate cirrhosis, liver failure, and HCC. Studies have shown that if primary care specialists are provided with guidelines for referring CHB patients to specialists, the number of patients receiving specialist care increases [51].

## 6. Lack of Diagnostic Facilities

CHB is an expensive disease to manage. Evaluation of a patient involves collecting a thorough history, detailed physical examination, and laboratory tests. These tests include serological viral markers, biochemical tests to assess hepatic injury, hormone assays, hematological tests, imaging studies, molecular biological studies (HBV DNA viral load assessment), and liver biopsy. These tests must be performed during initial assessment and treatment monitoring and at post-treatment follow-up. Many laboratories in Nigeria, including those in the teaching and specialist hospitals, can only conduct HBsAg screening. Other serological evaluations such as hepatitis B e antigen (HBeAg), hepatitis B e antibody (HBeAb), hepatitis B c antibody (HBcAb) (IgM, IgG), and hepatitis B s antibody (HBsAb) are often not conducted because of a lack of facilities and reagents. Currently, less than 6 centers in Nigeria are able to offer HBV DNA viral load measurements. Patients must travel hundreds of kilometers to such centers in order to undergo the test. The cost of these tests, particularly HBV DNA viral load assessment, is highly prohibitive. Few patients are able to complete all required tests for treatment initiation and monitoring. Testing and vaccination for hepatitis A virus (HAV) is standard practice for all patients with CHB infection, because HAV infection is recognized as a cause of flares in CHB. In most African countries, including Nigeria, there are typically no facilities for such testing and the vaccine is largely unavailable. Quality assurance in medical laboratories is a major issue in resource-poor countries. Prolonged power outages are frequent in Nigeria, which adversely affects reagents stored in refrigerators. CHB patients in Africa are largely HBeAg-negative. Therefore, absence of HBeAg should not limit the treatment of African patients. Most of what is known regarding HBV in Africans has been derived by extrapolation from studies done in Western countries and Asia. The exact duration of the immune tolerance phase   in Africans is not clearly known. Treatment of HBeAg-negative CHB patients is more difficult than treatment of HBeAg-positive CHB patients. Because of the high cost of virological tests, doctors frequently resort to liver biopsies to make treatment decisions.

## 7. Problems with Treatment

The most critical challenge in treating CHB is the high cost of medical care and antiviral drugs. The absence of an effective health insurance program with adequate coverage in Nigeria further compounds the problem. Patients must pay out-of-pocket at the point of service delivery, and very few persons are able to afford treatment. The result is that the majority of Nigerian patients diagnosed with CHB have not been properly evaluated and are not being treated. Pegylated interferon has a finite duration of treatment, and the patients do not become resistant. However, the cost of this treatment is not within the reach of most Nigerian patients. Monitoring which has to be instituted when a patient is on interferon therapy is another challenge. Frequent unpleasant side effects are responsible for discontinuation of the therapy in some patients. Nucleoside/nucleotide analogues are not as expensive as pegylated interferon, but the end-point of treatment has not been determined. In Nigeria, there is indiscriminate use of lamivudine because of lack of effective control. Some patients place themselves on this drug without proper evaluation. Thus, resistance to lamivudine is likely to be very high. Other nucleoside/nucleotide analogues (tenofovir, emtricitabine, and entecavir)  are very expensive and largely unavailable in Nigeria.

## 8. Co-Infections

### 8.1. HBV–HDV

The hepatitis D virus (HDV) is a defective virus with a circular RNA genome and a single structured protein, the hepatitis delta antigen. HBV has been shown to be important for HDV assembly and propagation. The distribution of HDV infection in Africa is not clearly known. In Nigeria, despite the availability of robust literature regarding the prevalence of HBV, there is little published work on HDV. Few studies have been published on HDV in Nigeria [52],[53],[54], and much remains to be understood regarding its distribution. HDV testing is not routinely available in most health institutions, including teaching hospitals. Thus, it is not usually factored into the management of patients with CHB, particularly, if hepatitis is present in the face of little or no HBV DNA (low viral load) in HBsAgpositive patients. Treatment of HDV infection requires interferon, which is expensive, requires close monitoring, and causes many significant side effects. More readily available and affordable nucleoside/nucleotide analogues are not used.

### 8.2. HBV–HCV

Infection with HBV and HCV may occur, as these two viruses share similar risk factors and transmission modes. As a consequence, co-infection with these two viruses occurs frequently, particularly in geographic areas where both viruses are endemic. Problems encountered in managing co-infected patients are considerable because two viruses are involved and viral load monitoring during treatment is necessary to determine which of them is dominant. This has obvious cost implications.

### 8.3.  HBV–HIV

Since HBV and HIV share common routes of transmission, the prevalence of hepatitis B markers (HBsAg and HBcAb) in HIV-infected patients is remarkably high. Around the world, 90% of HIV-infected persons have biological signs of prior HBV infection (defined by the presence of HBcAb) and 5–15% have a chronic infection (defined by the presence of serum HBsAg) [55]. In Africa, where HBV endemicity is high (>8%), most HBV infections occur within the first 5 years of life through close contact within households and medical or cultural procedures, e.g., scarification and tattoos [56]. In these settings, the prevalence of HBV infection is often close to 15%, regardless of HIV-co-infection [57]. In the era of broad-use of combined antiretroviral therapy (cART), the mortality and incidence of AIDS-defining diseases have dramatically declined [58]. Liver diseases, particularly those associated with HBV, have consequently emerged as a major cause of morbidity and mortality in HIV-infected persons [59][60][61]. Persistent HBeAg reactivity and persistently high levels of HBV DNA have been associated with increased progression of hepatitis B in HIV-co-infected persons[60]. Thus, therapeutic inhibition of HBV replication may be beneficial, even if only for liver disease evolution. However, the restoration of innate and adaptive immunity may be associated with flares of necroinflammatory activity in the liver [62]. There have also been instances of triple infection with HIV, HBV, and HCV [59] and quadruple infection with HIV, HBV, HCV, and HDV [63]. Similar combinations of infections may exist in resource-poor countries, but the necessary laboratory support for diagnosing and monitoring such patients is typically lacking. The availability of nucleoside/ nucleotide analogues with dual activity against HIV and HBV has initiated a transition in the paradigm of HBV treatment in the context of HIV, along with many advantages and drawbacks. The use of drug combinations (emticitabine/tenofovir) and of dual activity drugs (tenofovir, lamivudine, and emtricitabine) help to simplify both HIV and HBV management, but the onset of resistance warrants a strict viral follow-up. The incidence and spread of lamivudine-resistant and vaccine-escape HBV mutants is a major public health issue [57]. HBV and HCV screening is included as part of the initial clinical and biological guidelines recommended by the WHO when initiating antiretroviral therapy [64], but is not often applied in resource-poor countries due to financial constraints. In HBsAg-negative patients, immunization is strongly advised, but the late stages at which HIV infection is diagnosed and the cost of immunization which has to be borne by the patient limit efficacy in resource-poor countries. When HIV-infected patients are diagnosed with CHB, a first-line AIDS-associated retrovirus (ARV) regime should include lamivudine and tenofovir, as recommended by the WHO [65]. In resource-poor countries, access to tenofovir remains problematic, and if contraindicated, other treatment options are typically unavailable. Broader and cheaper access to HBV screening and first-line tenofovir-containing therapy should be advocated to prevent the emergence and spread of drugresistant strains [57][66]. Transmission of HBV from mother-to-child (HBV-MTCT) is prevented by administration of hepatitis B immunoglobulins (HBIgs). The high cost of HBIg makes its use in resource-poor settings a large problem. Furthermore, because HBV DNA levels are often very high during HBVHIV co-infection [67][68], the use of HBIg may also fail. Lamivudine is useful in preventing HBV-MTCT [63], but better results are obtained when combined with HBIg; however, this in not practical in resource-poor settings. Concern has also been raised with regard to the increasing prevalence of HCC following broader access to ARVs.Early diagnosis and treatment of HCC is unfortunately difficult in resource-limited settings. Other challenges faced not only by resource-poor countries but indeed the entire global community in the management of HBV infection include: Genotype and mutations of the HBV genome [63][67]. This problem is exacerbated because in HIV-HBV co-infection, long exposure to nucleoside/nucleotide analogues that have been largely used to treat HIV enhances the opportunity for mutations to occur. There is need for new viral markers in addition to HBV DNA. Treatment with potent drugs such as tenofovir leads to profound suppression of HBV DNA, but there is still a risk of developing cirrhosis and end-stage liver disease (ESLD). The covalently closed circular DNA (cccDNA) molecule is an interesting marker that may correlate better with treatment efficacy [69][70]. In addition, the plasma HBsAg concentration has been shown to be an accurate predictor of HBsAg seroconversion and HBV clearance [71]. In the context of HIV where nucleoside/ nucleotide analogues are more frequently used, HBsAg quantification may help tailor HBV treatment more precisely and assist in early detection of non-responding patients whose anti-HBV treatment may consequently be more rapidly maximized.

## 9. Way Forward

Health Education/Awareness Campaigns should be directed at the general populace and health care workers. For the general populace, it is important to understand HBV, how it is transmitted, its clinical course, and its complications. Specific high-risk practices such as body scarification, sharing of sharp instruments for body piercing, and unhealthy medical instrumentations should be discouraged. This is the best way to promote prevention measures. For health care workers, significant training must be conducted to increase their knowledge base and correct erroneous ideas regarding hepatitis B. Thorough screening of blood and blood products prior to transfusion must be ensured in all health institutions. Effective referral systems for the care of HBV-infected patients should be established since most laboratory tests and treatments require experts. Because of the large reservoir of asymptomatic infections in high prevalence areas, screening is the only way to identify these patients. People in these areas shou ld take advantage of opportunities for screening whenever they visit a hospital for any reason. Other occasions when screening can be instituted include prior to blood or organ donation, during school enrolment, military service, pre-employment, routine medical examination, and as part of medical insurance. Governments should make screening free and there should be an adequate management plan for those who test positive and vaccination of those who test negative. Existing childhood immunization programs should be intensified to improve coverage. The importance of this approach is illustrated by the experience in Taiwan [72]. Guidelines for primary care physicians should be published and existing ones should be updated to keep physicians informed on changes in CHB management. In regions in which there have been substantial investments in services to deliver HIV treatment (courtesy of donor agencies), existing facilities and staff could be expanded to incorporate HBV mono-infected individuals. As done in case of HIV/AIDS, efforts can be devoted to negotiating affordable drugs and establishing reliable supply chains. Pharmaceutical companies that produce anti-HBV drugs or HBV assays should lower the price of their products. Companies should provide support in the form of research/ educational grants and continuing medical education programs for primary care physicians, internists, and gastroenterologists in capacity building. Technical and financial assistance should be provided by wealthier nations of the world for screening, monitoring, and drug treatment of CHB. All recommendations of the Global Hepatitis Policy should be immediately implemented. Health insurances should be strengthened in scope and content, e.g., diseases such as CHB should be included. An adequate number of laboratories should be established and located in such a way that patients will not have to travel long distances for laboratory tests. More hepatologists, pathologists, and allied staff should be trained. Centers of excellence for Liver/Digestive diseases should be established. In such centers, resources can be pooled for effective service delivery, research, and training. Such centers will aim at starting liver transplant programs in reasonable time.

## References

[1] Lavanchy D (2004). Hepatitis B virus epidemiology, disease burden, treatment, and current and emerging prevention and control measures. J Viral Hepat.

[2] Murray CJ, Lopez AD (1997). Mortality by cause for eight regions of the world: Global Burden of Disease Study. Lancet..

[3] (2004). Hepatitis B vaccines. Wkly Epidemiol Rec.

[4] Kramvis A, Kew MC (2007). Epidemiology of hepatitis B virus in Africa, its genotypes and clinical associations of genotypes. Hepatol Res.

[5] Merican I, Guan R, Amarapuka D, Alexander MJ, Chutaputti A, Chien RN, Hasnian SS, Leung N, Lesmana L, Phiet PH (2000). Chronic hepatitis B virus infection in Asian countries. J Gastroenterol Hepatol.

[6] Vardas E, Mathai M, Blaauw D, McAnerney J, Coppin A, Sim J (1999). Preimmunization epidemiology of hepatitis B virus infection in South African children. J Med Virol.

[7] Beasley RP, Trepo C, Stevens CE, Szmuness W (1977). The e antigen and vertical transmission of hepatitis B surface antigen. Am J Epidemiol.

[8] Roingeard p, Diouf A, Sankale JL, Boye C, Mboup S, Diadhiou F, Essex M (1993). Perinatal transmission of hepatitis B virus in Senegal, west Africa. Viral Immunol.

[9] The World Bank. World Development Indicators. Nigeria: Data and statistics. http://go.worldbank.org/HREMVJ8T90..

[10] Idoko J, Meloni S, Muazu M, Nimzing L, Badung B, Hawkins C, Sankalé JL, Ekong E, Murphy R, Kanki P, Thio CL (2009). Impact of Hepatitis B Virus Infection on Human Immunodeficiency Virus Response to Antiretroviral Therapy in Nigeria. Clin Infect Dis.

[11] Abiodun PO, Ihongbe JC, Ubaru RJ (1985). HBs antigen and blood donors in Benin City, Nigeria. East Afr Med J.

[12] Bada AS, Olatunji PO, Adewuyi JO, Iseniyi JO, Onile BA (1996). Hepatitis B surface antigenaemia in Ilorin, Kwara State, Nigeria. Cent Afr J Med.

[13] Fakunle YM, Ajdukiewicz AB, Greenwood BM, Edington GM (1977). Primary liver cell carcinoma (PLCC) in the Northern Guinea Savanna of Nigeria. Trans R Soc Trop Med Hyg.

[14] Mustapha SK, Pindiga UH (2004). The Prevalence of Hepatitis B Virus in Patients With Hepatocellular Carcinoma in Gombe, North Eastern Nigeria. Sahel Med J.

[15] Ndububa DA, Ojo OS, Adeodu OO, Adetiloye VA, Olasode BJ, Famurewa OC, Durosinmi MA, Agbakwuru AE (2001). Primary hepatocellular carcinoma in Ile-Ife, Nigeria: a prospective study of 154 cases. Niger J Med.

[16] Nwokediuko S, Ijoma UN (2011). Relatively High Seroprevalence of Hepatitis B Surface Antigen in Female Civil Servants in Enugu State of Nigeria Sylvester Chuks Nwokediuko, Uchenna Ijoma. Euroasian J Hepato- Gastroenterol [Internet]..

[17] Nwokediuko S, Ijoma UN, Obienu O (2011). Liver Cancer in Enugu, South East Nigeria. Insight. 2011;1(1):1-5.

[18] Oli JM, Okafor GO (1980). The prevalence of hepatitis B surface antigen in Nigerian diabetics. Trop Geogr Med.

[19] Olubuyide IO, Aliyu B, Olalelye OA, Ola SO, Olawuyi F, Malabu UH, Odemuyiwa SO, Odaibo GN, Cook GC (1997). Hepatitis B and C virus and hepatocellular carcinoma. Trans R Soc Trop Med Hyg.

[20] Otu AA (1987). Hepatocellular carcinoma, hepatic cirrhosis, and hepatitis B virus infection in Nigeria. cancer.

[21] Sirisena ND, Njoku MO, Idoko JA, Isamade E, Barau C, Jelpe D, Zamani A, Otowo S (2002). Carriage rate of hepatitis-B surface antigen (HBsAg) in an urban community in Jos, Plateau State, Nigeria. Niger Postgrad Med J.

[22] Beasley RP (2009). Rocks along the road to the control of HBV and HCC. Ann Epidemiol.

[23] Mast E, Mahoney F, Kane M, Margolis HS, Plotkin SA Orenstein WA (2004). Hepatitis B vaccine. Infectious Diseases Related To Travel.

[24] Sadoh AE, Eregie CO (2008). Age at presentation for infant immunization in Nigeria: Implications for hepatitis B immunization. Public health.

[25] (2005). World Health Organization. vaccine preventable diseases: monitoring system. 2006 global summary. Immunization Vaccines and Biologicals. http://www.who.int/vaccines-documents/GlobalSummary/GlobalSummary.pdf.

[26] Amazigo UO, Chime AB (1990). Hepatitis-B virus infection in rural and urban populations of eastern Nigeria: prevalence of serological markers. East Afr Med J.

[27] Mutimer DJ, Olomu A, Skidmore S, Olomu N, Ratcliffe D, Rodgers B, Mutimer HP, Gunson BK, Elias E (1994). Viral hepatitis in Nigeria--sickle-cell disease and commercial blood donors. QJM.

[28] Obiaya M, Ebohom PA (1982). Blood Transfusion Hazards in Benin City, Nigeria. Nig Med J. 1982; 12:251-4.

[29] Jayaraman S, Chalabi Z, Perel P, Guerriero C, Roberts I (2010). The risk of transfusion-transmitted infections in sub-Saharan Africa. Transfusion.

[30] Centre for Disease control and prevention (CDC). Division of viral hepatitis B. http://www.cdc.gov/ncidod/diseases/none%20hepatitis/b/fact.

[31] Nwokediuko S (2010). Risk factors for Hepatitis B virus transmission in Nigerians: A case-control study. The Internet Journal of Gastroenterology.

[32] Ivlustapha SK, Jibrin YB (2004). The Prevalence of Hepatitis B Surface Antigenemia in Patients with Human Immunodeficiency Virus infection in Gombe, Nigeria. Ann Afrii Med.

[33] Norder H, Hammas B, Lee SD, Bile K, Courouce AM, Mushahwar IK, Magnius LO (1993). Genetic relatedness of hepatitis B viral strains of diverse geographical origin and natural variations in the primary structure of the surface antigen. J Gen Virol.

[34] Okamoto H, Tsuda F, Sakugawa H, Sastrosoewignjo RI, Imai M, Miyakawa Y, Mayumi M, Miyakawa Y, (1988). Typing Hepatitis B Virus by Homology in Nucleotide Sequence: Comparison of Surface Antigen Subtypes. J Gen Virol.

[35] Stuyver L, De Gendt S, Van Geyt C, Zoulim F, Fried M, Schinazi R, Rossau R (2000). A new genotype of hepatitis B virus: complete genome and phylogenetic relatedness. J Gen Virol.

[36] Mulders MN, Venard V, Njayou M, Edorh AP, Bola Oyefolu AO, Kehinde MO, Muyembe Tamfum JJ, Nebie YK, Maiga I, Ammerlaan W, Fack F, Omilabu SA, Le Faou A, Muller CP (2004). Low genetic diversity despite hyperendemicity of hepatitis B virus genotype E throughout West Africa. J Infect Dis. 2004;190(2):400.

[37] Olinger CM, Venard V, Njayou M, Oyefolu AO, Maïga I, Kemp AJ, Omilabu SA, le Faou A, Muller CP (2006). Phylogenetic analysis of the precore/core gene of hepatitis B virus genotypes E and A in West Africa: new subtypes, mixed infections and recombinations. J Gen Virol.

[38] Forbi JC, Vaughan G, Purdy MA, Campo DS, Xia GL, Ganova-Raeva LM, Ramachandran S, Thai H, Khudyakov YE (2010). Epidemic history and evolutionary dynamics of hepatitis B virus infection in two remote communities in rural Nigeria. PLoS One.

[39] Isiugo-Abanihe UC (2006). Sociocultural aspects of HIV/AIDS infection in Nigeria. Afr J Med Med Sci.

[40] Uwaezuoke SN, Nneli RO (2007). eath of a G-6-P-D deficient child with co-morbid HIV infection linked to scarification. J Trop Paediatr.

[41] Drucker E, Alcabes PG, Marx PA (2001). 41. Drucker E, Alcabes PG, Marx PA. The injection century: massive unsterile injections and the emergence of human pathogens. Lancet.

[42] Foege WH, Eddins DL (1973). Mass vaccination programs in developing countries. Prog Med Virol.

[43] Foege WH, Millar JD, Henderson DA (1975). Smallpox eradication in West and Central Africa. Bull World Health Organ.

[44] Simonsen L, Kane A, Lloyd J, Zaffran M, Kane M (1999). Unsafe injections in the developing world and transmission of bloodborne pathogens: a review. Bull World Health Organ.

[45] Parkin DM, Sitas F, Chirenje M, Stein L, Abratt R, Wabinga H, Part I (2008). Cancer in Indigenous Africans--burden, distribution, and trends. Lancet Oncol.

[46] World Hepatitis Alliance. Viral Hepatitis: Global Policy. http://www.hivandhepatitis.com/2010¬¬_conference/easl/docs/0420¬_2010_a.html.

[47] Lok ASF, McMahon BJ (2001). Chronic hepatitis B. Hepatology.

[48] Mullan F (2005). The metrics of the physician brain drain. N Engl J Med.

[49] Ihekweazu C, Anya I, Anosike E (2005). Nigerian medical graduates: where are they now?. Lancet.

[50] Castera L, Forns X, Alberti A (2008). Non-invasive evaluation of liver fibrosis using transient elastography. J Hepatol.

[51] Mostert MC, Richardus JH, de Man RA (2004). Referral of chronic hepatitis B patients from primary to specialist care: making a simple guideline work. J Hepatol.

[52] Nwokediuko SC, Ijeoma U (2009). Seroprevalence of antibody to HDV in Nigerians with hepatitis B virus-related liver diseases. Niger J Clin Pract..

[53] Ojo OS, Akonai AK, Thursz M, Ndububa DA, Durosinmi MA, Adeodu OO, Fatusi OA, Goldin RD (1998). Hepatitis D virus antigen in HBsAg positive chronic liver disease in Nigeria. East Afr Med J.

[54] Ojo OS, Thursz M, Thomas HC, Ndububa DA, Adeodu OO, Rotimi O, Lawal AA, Durosinmi MA, Akonai AK, Fatusi AO (1995). Hepatitis B virus markers, hepatitis D virus antigen and hepatitis C virus antibodies in Nigerian patients with chronic liver disease. East Afr Med J.

[55] Alter MJ (2006). Epidemiology of viral hepatitis and HIV co-infection. J Hepatol.

[56] Adewole OO, Anteyi E, Ajuwon Z, Wada I, Elegba F, Ahmed P, Betiku Y, Okpe A, Eze S, Ogbeche T, Erhabor GE (2009). Hepatitis B and C virus co-infection in Nigerian patients with HIV infection. J Infect Dev Ctries.

[57] Hoffmann CJ, Thio CL (2007). Clinical implications of HIV and hepatitis B co-infection in Asia and Africa. Lancet Infect Dis.

[58] Palella FJ Jr, Baker RK, Moorman AC, Chmiel JS, Wood KC, Brooks JT, Holmberg SD (2006). Mortality in the highly active antiretroviral therapy era: changing causes of death and disease in the HIV outpatient study. J Acquir Immune Defic Syndr.

[59] Konopnicki D, Mocroft A, de Wit S, Antunes F, Ledergerber B, Katlama C, Zilmer K, Vella S, Kirk O, Lundgren JD (2005). Hepatitis B and HIV: prevalence, AIDS progression, response to highly active antiretroviral therapy and increased mortality in the EuroSIDA cohort. Aids.

[60] Lewden C, May T, Rosenthal E, Burty C, Bonnet F, Costagliola D, Jougla E, Semaille C, Morlat P, Salmon D, Cacoub P, Chêne G (2008). Changes in causes of death among adults infected by HIV between 2000 and 2005: The” Mortalite 2000 and 2005” surveys (ANRS EN19 and Mortavic).. J Acquir Immune Defic.

[61] Mocroft A, Brettle R, Kirk O, Blaxhult A, Parkin JM, Antunes F, Francioli P, D'Arminio Monforte A, Fox Z, Lundgren JD (2002). Changes in the cause of death among HIV positive subjects across Europe: results from the EuroSIDA study. Aids.

[62] Puoti M, Torti C, Bruno R, Filice G, Carosi G (2006). Natural history of chronic hepatitis B in co-infected patients. J hepatol.

[63] Lacombe K, Massari V, Girard PM, Serfaty L, Gozlan J, Pialoux G, Mialhes P, Molina JM, Lascoux-Combe C, Wendum D, Carrat F, Zoulim F (2006). Major role of hepatitis B genotypes in liver fibrosis during coinfection with HIV. Aids.

[64] (2008). Essential prevention and care interventions for adults and adolescents living with HIV in resource- limited settings. Switzerland: WHO. 2008. World Health Organization.

[65] Priority Interventions. HIV/AIDS prevention and care in the health sector. World Health Organization.

[66] Lessells RJ, Main J, Cooke GS (2008). HIV and hepatitis B co-infection in Africa. Lancet Infect Dis.

[67] Liu SL, Dong Y, Zhang L, Li MW, Wo JE, Lu LW, Chen ZJ, Wang YZ, Ruan B (2009). Influence of HBV gene heterogeneity on the failure of immunization with HBV vaccines in eastern China. Arch Virol.

[68] Wiseman E, Fraser MA, Holden S, Glass A, Kidson BL, Heron LG, Maley MW, Ayres A, Locarnini SA, Levy MT (2009). Perinatal transmission of hepatitis B virus: an Australian experience. Med J Aust.

[69] Chan HLY, Tse CH, Mo F, Koh J, Wong VWS, Wong GLH, Lam Chan S, Yeo W, Sung JJ, Mok TS (2008). High viral load and hepatitis B virus subgenotype ce are associated with increased risk of hepatocellular carcinoma. J clin oncol.

[70] Werle-Lapostolle B, Bowden S, Locarnini S, Wursthorn K, Petersen J, Lau G, Trepo C, Marcellin P, Goodman Z, Delaney WE 4th, Xiong S, Brosgart CL, Chen SS, Gibbs CS, Zoulim F (2004). Persistence of cccDNA during the natural history of chronic hepatitis B and decline during adefovir dipivoxil therapy. Gastroenterology.

[71] Brunett MR, Moriconi F, Bonino F, Lau GK, Farci P, Yurdaydin C, Piratvisuth T, Luo K, Wang Y, Hadziyannis S, Wolf E, McCloud P, Batrla R, Marcellin P (2009). Hepatitis B virus surface antigen levels: a guide to sustained response to peginterferon alfa-2a in HBeAg-negative chronic hepatitis B. Hepatology.

[72] Chang MH, Chen CJ, Lai MS, Hsu HM, Wu TC, Kong MS, Liang DC, Shau WY, Chen DS (1997). Universal hepatitis B vaccination in Taiwan and the incidence of hepatocellular carcinoma in children. Taiwan Childhood Hepatoma Study Group. New Engl J Med.

